# Probing heart rate variability to determine parasympathetic dysfunction

**DOI:** 10.14814/phy2.13713

**Published:** 2018-05-23

**Authors:** Jan D. Huizinga, Karen J. Mathewson, Yuhong Yuan, Ji‐Hong Chen

**Affiliations:** ^1^ Department of Medicine Farncombe Family Digestive Health Research Institute McMaster University Hamilton Ontario Canada; ^2^ Department of Psychology Neuroscience and Behaviour McMaster University Hamilton Ontario Canada

Dear Editor,

We read with interest the recent paper by Haraldsdottir et al. (2018) which used measures of heart rate variability in both time and frequency domain to assess autonomic dysfunction in adolescents that were born prematurely.

The conclusion of the paper is that adolescent children with a history of very premature birth have autonomic dysfunction and are therefore at elevated risk of later cardiovascular disease. It is probably always good to be aware of risk factors. On the other hand, without strong evidence, one should not label healthy individuals as having an increased risk of a possibly fatal disease.

The authors emphasize several measures of cardiac parasympathetic reactivity in the time domain, RMSSD (Root Mean Square of Successive RR Differences) and SDRR (Standard deviation of RR intervals), where are RR is the interval between successive peaks of the QRS complex. They show that the average values of RMSSD and SDRR in adolescents born very prematurely are significantly lower than the average values of a group of term‐born controls. They come to the conclusion that “the lower time domain variability as expressed by the SDRR and RMSSD in preterm individuals is indicative of blunted autonomic activity” and “a lower RMSSD is correlated with lower parasympathetic activity.” They then formulate their end conclusion as “children born preterm ages 12–14 exhibit abnormal autonomic function, consistent with a re‐emergence of disease”.

The major problem with this conclusion is that the authors also measure the “gold standard” of parasympathetic reactivity from the frequency domain, respiratory sinus arrhythmia (RSA) (Bonaz et al. 2016). In Haraldsdottir et al. (2018), RSA is termed HF(ms^2^), but the values express are ln(ms^2^). The RSA values do not show a significant difference between the preterm and the term‐born children. In fact, the values show a trend toward **increased** parasympathetic reactivity in the preterm children. The authors omit this critically important aspect of their study in their discussion, yet the RSA data should be interpreted such that preterm adolescents do NOT have abnormal autonomic sympathetic reactivity.

It should be mentioned that it is correct that their RMSSD values show a statistically significant difference that suggests decreased parasympathetic activity in preterm children, but most values pertaining to the children in the preterm group are within the range of normal when compared to their own control group. Hence, the statistical difference does not appear to translate into a physiologically significant difference for almost all the children.

The heart rate recovery (HRR) was shown to be significantly lower in the preterm group compared to the term‐born group. But again, this does not have clinical meaning since the values in the preterm group are within normal limits, based on data from 485 children (Singh et al. 2008). One cannot derive a normal range of values from a control group of 13 children. Therefore, the HRR values also do not provide any evidence of autonomic dysfunction in the preterm group.

We believe that it is important to point out to that it is premature to label preterm adolescents as having a higher than normal chance of cardiovascular disease.

## Conflict of Interest

The authors declare that there is no conflict of interest.
